# Club cell protein 16 and cytokeratin fragment 21-1 as early predictors of pulmonary complications in polytraumatized patients with severe chest trauma

**DOI:** 10.1371/journal.pone.0175303

**Published:** 2017-04-05

**Authors:** Lukas L. Negrin, Gabriel Halat, Stephan Kettner, Markus Gregori, Robin Ristl, Stefan Hajdu, Thomas Heinz

**Affiliations:** 1 Department of Trauma Surgery, Medical University of Vienna, Vienna, Austria; 2 Department of Anesthesiology, General Intensive Care and Pain Management, Medical University of Vienna, Vienna, Austria; 3 Center for Medical Statistics and Informatics, Medical University of Vienna, Vienna, Austria; University of Florida, UNITED STATES

## Abstract

**Background:**

Acute respiratory distress syndrome (ARDS) and pneumonia have a great impact on the treatment regimen of polytraumatized patients with severe chest trauma. The objective of our study was to determine whether biomarker levels assessed shortly after multiple trauma may predict the occurrence of these conditions.

**Methods and findings:**

Our patient population included 71 men and 30 women (mean age, 40.3 ± 15.8 years) with an Injury Severity Score that ranged from 17 to 59 and an Abbreviated Injury Scale Thorax of at least 3. They were admitted to our level I trauma center within one post-traumatic hour and survived for at least 24 hours after the trauma occurred. Thirty-five patients developed ARDS, 30 patients pneumonia and 21 patients both. Five individuals died during hospitalization. The levels of five selected biomarkers, which were identified by a literature search, were assessed at admission (initial levels) and on day 2 after trauma. We performed comparisons of medians, logistic regression analyses and receiver operating characteristic analyses for initial and day-2 levels of each biomarker. With regard to ARDS, initial levels of cytokeratin fragment 21–1, the soluble fragment of cytokeratin 19 (CYFRA21-1) and of the club cell protein 16 (CC16) provided significant results in each statistical analysis. With regard to pneumonia, each statistical analysis supplied significant results for both initial and day-2 levels of CYFRA21-1 and CC16. Consistently, initial CYFRA21-1 levels were identified as the most promising predictor of ARDS, whereas day-2 CC16 levels have to be considered as most appropriate for predicting pneumonia.

**Conclusions:**

CYFRA21-1 levels exceeding cut-off value of 1.85 ng/ml and 2.49 ng/ml in the serum shortly after multiple injury occurred may identify polytraumatized patients at risk for ARDS and pneumonia, respectively. However, CC16 levels exceeding 30.51 ng/ml on day 2 may allow a firmer diagnosis for the development of pneumonia.

## Introduction

Acute respiratory distress syndrome (ARDS) and pneumonia are common complications in multiply injured patients, particularly in those with chest injuries [1–3]. Independent risk factors for the development of trauma-related ARDS are an ISS higher than 25 [4], the presence of a lung contusion, a transfusion requirement of more than 10 units within 24 hours, hypotension on admission, and an age over 65 years [5]. ARDS following chest trauma is caused by a bruise to the thorax that predominately results in local severe disruption of the lung epithelium and subsequently, to a minor extent, also in a damage of the vascular endothelium [6, 7]. The integrity of the alveolar-capillary barrier is destroyed, leading to the formation of additional protein-rich alveolar edema, which provoke extensive activation of innate inflammatory responses. Due to the loss of aerated lung tissue, clinical deterioration progresses [8–10]. Although a low mortality rate of 24.1% has been reported in patients suffering a severe trauma-related ARDS [11], their treatment and rehabilitation poses a great socio-economic burden [12]. Even two years after hospital discharge, persistent functional disability and impaired quality of life have to be expected in ARDS survivors [13]. Therefore, early identification and continuous monitoring of patients at risk for developing ARDS, as well as implementing prevention strategies early after admission, are key factors in decreasing its occurrence and optimizing therapy. Pneumonia is caused by microbial agents from damaged mucosal surfaces that invade the lung parenchyma, provoking intra-alveolar exudates [14, 15]. Independent risk factors of pneumonia are lung contusion, hematothorax, the need and duration of mechanical ventilation [16, 17], re-intubation, supine position, advanced age [18], the number of antibiotics received in the past [19], and obesity [20]. ARDS and pneumonia are closely connected. Whereas ARDS is often complicated by ventilator-associated pneumonia in trauma patients, nosocomial pneumonia has been identified as the most frequent single cause of ARDS in non-trauma patients [21].

As a result of diffuse alveolar damage [22] and increased capillary permeability, cytokines are supposed to be released in the circulation [23]. We hypothesized that, after mechanical damage to the lung epithelial barrier caused by trauma, cytokines might also be secreted into the serum and that they might be capable of predicting the development of ARDS and/or pneumonia by increased or decreased levels, assessed directly at, or several hours after, admission. These biomarkers might be helpful in clinical practice to plan surgery and preventive treatment of lung injury in patients at risk for ARDS. Elevated levels of the soluble secretory isoform of the receptor for advanced glycation end products (sRAGE) [24, 25], of the club cell protein 16 (CC16) [26, 27], of the surfactant protein D (SP-D) [28], and of Krebs von den Lungen 6/Mucin 1 (KL-6/MUC1) [29] have already been identified in the blood of patients suffering ARDS. Moreover, elevated concentrations of cytokeratin fragment 21–1, the soluble fragment of cytokeratin 19 (CYFRA21-1) [30], have been found in bronchoalveolar lavage (BAL) fluid of these patients. Accordingly, we determined serum levels of sRAGE, CC16, SP-D, CYFRA21-1, and KL-6/MUC1 in polytraumatized patients with severe chest trauma at risk for developing ARDS and/or pneumonia at admission and on the second day after the trauma occurred.

## Material and methods

Our prospective observational study, approved by the Ethics Committee of the Medical University of Vienna (project number 368/2011), was performed over a period of four years. The inclusion criteria were (1) polytraumatized patients (ISS≥16); (2) with an AIS_Thorax_ ≥3; (3) who were at least 18 years old; (4) who were directly admitted to our level I trauma center within one hour after injury; (5) who were transferred to the intensive care unit (ICU) after initial treatment due to their life-threatening condition; and (6) who survived their injury for at least 24 hours. Burn victims and patients with a known history of malignancies, inflammatory diseases or other lung disorders were excluded. In order to provide comparative values, a control group of 10 healthy individuals was frequency matched according to age and gender. ARDS was diagnosed according to the Berlin definition [10]. Clinical evidence for the diagnosis of pneumonia included an abnormal temperature (> 38°C or < 35.5°C), either leucocytosis (white cell count > 10,000/mm3 or > 10% immature forms) or leucopenia (white cell count < 4,000/mm3), a macroscopically purulent sputum, the presence of a new cough, dyspnea, and/or tachypnea (in the case of spontaneous breathing patients) as well as a new or changing infiltrate on chest radiograph. Empiric antibiotic therapy was started immediately. All intubated patients with clinical symptoms of pneumonia underwent fiber-optic bronchoscopy with BAL. A quantitative culture was obtained. If the BAL effluent contained ≥ 10^5^ organisms/ml, the patient was diagnosed with pneumonia and intravenous antibiotics were continued.

### Blood sampling and analysis

During initial assessment and diagnostics, together with the routine venous blood samples, one separating gel tube (Vacuette^®^ 4 ml; Greiner Bio-One International) was withdrawn from each polytraumatized patient for biomarker level measurement. Immediately afterwards, this additional sample was centrifuged at 3,000g for 15 minutes at room temperature and stored at -80°C until the patient or his/her family members could be asked for an informed consent. If written consent was not given, the serum was disposed of. Otherwise, it was stored further at -80°C until assayed. In study participants, blood samples for biomarker level measurement were taken again on day 2, therefore 24 to 48 hours after trauma, dependent on clinical routine and staff resources. According to the instructions, ELISA-Kits were used to assess levels of sRAGE *(Human RAGE Immunoassay*, *Quantikine*^®^ *ELISA; R&D Systems*, *Inc; Cat*.*Nr*.: *DRG00*, *SRG00*, *PDRG00)*, of CC16 *(Human Uteroglobin Immunoassay*, *Quantikine*^®^ *ELISA; R&D Systems*, *Inc; Cat*.*Nr*.: *DUGB00)*, of SP-D *(Human SP-D Immunoassay*, *Quantikine*^®^ *ELISA; R&D Systems*, *Inc; Cat*.*Nr*.: *DSFPD0)*, of CYFRA21-1 *(Human TM-CYFRA 21–1 ELISA Kit; DRG International Inc*.*; Cat*.*Nr*.: *EIA-5070)*, and of KL-6/MUC-1 *(Human Kl-6/MUC1 ELISA Kit; BlueGene Biotech CO*., *LTD; Cat*.*Nr*.: *E01K0061)*. All samples were analyzed in triplets and the mean values were calculated.

After primary care in the trauma unit, all patients were transferred to the ICU. Standardized treatment included lung protective ventilation according to the recommendations of the ARDS network [31], restriction of fluid management after initial resuscitation, and prone positioning, if feasible. All relevant information was processed to the routine patient data management system of our department.

### Statistical analysis

Statistical analysis was performed using IBM SPSS Statistics Version 23, 64 bit. Symmetrically distributed parameters are presented as mean and standard deviation. For skew distributions, parameters are displayed as median and interquartile range in round brackets, for ordinal categorical data median and range in square brackets are indicated. Student’s t-tests were used to compare symmetrically distributed parameters, whereas continuous variables of a skew distribution were compared by means of the Mann-Whitney-Wilcoxon rank-sum test (for unrelated samples) and the Wilcoxon signed-rank test (for related samples). The Chi-square test was applied to analyze categorical data. Due to the fact that the outcome of interest was a binary variable (“occurrence of ARDS” and “occurrence of pneumonia”, respectively), logistic regression analyses were performed. The distributions of the considered predictors were highly right skewed with some large outliers. To allow for reliable estimation, all variables were log-transformed before being used as independent variables. Therefore, odds ratios (OR) with 95% confidence interval (CI) are presented at the log-scale. With regard to single logistic regression analyses, both the initial and the day-2 level of all five biomarkers chosen defined the relevant single independent (predictor) variable. In order to incorporate the change in biomarker levels, the quotient formed by the day-2 level divided by the corresponding initial level of each biomarker was also used as independent variable. Additionally, we performed multiple logistic regression analyses, depending on the common predictor variables “initial level”, “day-2 level”, “age”, and “ISS” for each biomarker, as well as multiple logistic regression analyses including several biomarker levels as independent variables. For receiver operating characteristic (ROC) curves, the area under the curve (AUC) was calculated and indicated with a 95% CI. It is noteworthy that ROC analysis is essentially non-parametric, not affected by the skewed nature of the distributions, and invariant to log-transformation. Cut-off values were determined by the maximum sum of sensitivity and specificity [32]. In general, 0.05 was set as level of significance.

## Results

From 2011 to 2015 101 patients (71 men and 30 women) with a mean age of 40.3 ± 15.8 [18–82] years—men, 39.9 ±15.1 years; women, 41.2 ± 17.4 years—and a mean ISS of 33.7 ± 11.3 [17–59] met the inclusion criteria. Fifty-seven patients were transported overland to our trauma center and 58 were flown in by a rescue helicopter. Motor vehicle accident was the most common cause of injury (44.6%), followed by falls from a height of at least three meters (23.8%) and pedestrian/vehicle collisions (22.8%). Fifty-five trauma victims (54.5%) were admitted with orotracheal intubation, 15 trauma victims (14.9%) were already treated with a chest tube on the site of injury and 41 additionally at the trauma resuscitation room. Thoracic injury included parenchymal lung injury (78.2%), pneumothorax (47.5%), hematothorax (8.9%), hematopneumothorax (7.9%), rib fractures (80.2%), flail chest (20.8%), sternum fracture (19.8%), thoracic spine fractures (29.6%), extensive surgical emphysema (19.8%), and aortal dissection (6.9%). An AIS_Thorax_ of 3 was diagnosed in 40 trauma victims (39.6%), an AIS_Thorax_ of 4 in 35 (34.7%) and an AIS_Thorax_ of 5 in 26 trauma victims (25.7%). Five patients died during hospitalization after a time period of median 3 [1–24] post-traumatic days. The cause of death was ARDS in three patients, multiple organ failure not provoked by ARDS in one patient, and traumatic brain injury in one patient. All five non-survivors had arrived orotracheal intubated, presenting an AIS_Thorax_ of 4 or 5 and thus a higher mean ISS than the survivor group (51.2 versus 32.7; p = 0.0002).

Thirty-five patients (34.7%) developed ARDS, including 21 individuals who subsequently suffered pneumonia. Additionally, pneumonia was diagnosed in nine individuals without ARDS. Eighty-five patients (84.2%) needed mechanical ventilation during their stay at the ICU. Of interest, all patients who developed ARDS and/or pneumonia were transferred to the ICU under mechanical ventilation. As Table 1 displays, the levels of each biomarker changed significantly within the first two post-traumatic days. Furthermore, at both time points, biomarker levels of polytraumatized patients differed remarkably from those of a healthy control group. The latter included seven men and three women with a mean age of 38.7 years and 42.2 years respectively who did not undergo any treatment and did not suffer medical conditions over a period of several weeks prior to blood sampling.

**Table 1:** Overall biomarker levels presented as median and interquartile range.

	At admission	Post-injury day-2day-2	p	Healthy control
**sRAGE (pg/ml)**	2918 (1595–6943)	1429 (958–2585)	<0.0001	843 (608–1398)
**CC16 (ng/ml)**	41.02 (24.19–64.71)	25.85 (17.83–43.59)	<0.0001	13.50 (10.39–16.81)
**SP-D (ng/ml)**	5.91 (3.10–9.67)	8.18 (4.39–12.33)	<0.0001	9.16 (5.41–18.05)
**CYFRA21-1 (ng/ml)**	1.66 (1.14–2.67)	0.94 (0.56–1.53)	<0.0001	0.76 (0.53–1.12)
**KL-6/MUC1 (U/ml)**	2.20 (1.19–3.33)	2.64 (1.81–3.37)	0.017	1.21 (0.29–1.69)

The p-values refer to the comparison of biomarker levels assessed at admission and on day 2.

### ARDS

Ten, 21 and four patients developed mild, moderate and severe ARDS respectively, after a median of 2 (1–2) post-traumatic days. Not surprisingly, patients developing ARDS presented with a higher overall and higher thoracic injury severity and they had to be ventilated longer compared to those without ARDS, resulting in a longer length of stay (LOS) in the ICU. Pre-hospital intubation and pre-hospital chest-tube insertion have been identified as significant risk factors for ARDS (Table 2).

**Table 2:** Demographic data with regard to ARDS and pneumonia.

	ARDS			Pneumonia		
	Yes	No	p	Yes	No	p
Number	35	66		30	71	
Deceased	3	2	0.222	1	4	0.626
Age (years)	42.9 ± 15.0	38.8 ± 16.1	0.218	44.8 ± 16.7	38.8 ± 15.1	0.059
ISS	**38.4 ± 11.0**	**31.7 ± 10.6**	**0.002**	**38.6 ± 9.9**	**31.6 ± 11.2**	**0.004**
AIS_Thorax_	**4 [3–5]**	**3 [3–5]**	**0.0002**	4 [3–5]	4 [3–5]	0.224
Sex (male: female)	27:8	44:22	0.273	48:23	48:23	0.362
Prehospital intubation	**80.0%**	**40.9%**	**0.0002**	**83.3%**	**42.3%**	**0.0002**
Prehospital chest-tube	**28.6%**	**7.6%**	**0.005**	**33.3%**	**7.0%**	**0.001**
Chest tube trauma roomroom	40.0%	40.9%	0.929	33.3%	43.7%	0.334
Mechanical ventilation (days)	**10 (4–19)**	**2 (1–6)**	**<0.0001**	**14 (8–25)**	**2 (1–4)**	**<0.0001**
ICU LOS (days)	**16 (8–25)**	**8 (2–14)**	**<0.0001**	**21 (14–32)**	**6 (2–12)**	**<0.0001**
Overall LOS (days)	31 (17–74)	24 (17–46)	0.107	**45 (30–77)**	**22 (13–38)**	**<0.0001**
sRAGE (pg/ml)						
At admission	3956 (1662–7553)	2631 (1317–5454)	0.169	2782 (1541–4775)	2966 (1608–7244)	0.571
Post-injury day-2	1623 (1119–3884)	1408 (942–1906)	0.064	1571 (1057–3055)	1406 (943–2441)	0.236
CC16 (ng/ml)						
At admission	**51.85 (27.03–89.81)**	**39.56 (23.53–50.60)**	**0.038**	**56.47 (30.53–85.82)**	**39.21 (22.74–49.47)**	**0.002**
Post-injury day-2	36.83 (18.63–45.67)	23.60 (16.72–42.90)	0.340	**41.22 (24.41–64.09)**	**23.39 (16.09–37.57)**	**0.001**
SP-D (ng/ml)						
At admission	5.93 (3.05–9.10)	5.55 (2.94–10.03)	0.997	4.80 (2.62–7.78)	6.92 (3.14–10.04)	0.230
Post-injury day-2	9.00 (4.61–15.32)	7.62 (3.82–11.30)	0.288	8.03 (4.01–12.10)	8.18 (4.39–12.76)	0.699
CYFRA21-1 (ng/ml)						
At admission	**2.42 (1.27–4.46)**	**1.51 (1.07–2.25)**	**0.005**	**2.51 (1.39–5.22)**	**1.52 (1.05–2.27)**	**0.003**
Post-injury day-2	0.97 (0.68–1.72)	0.91 (0.52–1.53)	0.483	**1.28 (0.77–1.74)**	**0.87 (0.42–1.43)**	**0.018**
KL6/MUC-1 (U/ml)						
At admission	2.73 (1.65–3.50)	1.89 (1.15–3.11)	0.286	2.55 (1.78–2.62)	2.05 (1.13–3.63)	0.959
Post-injury day-2	2.66 (1.75–3.77)	2.48 (1.85–3.63)	0.902	2.64 (1.68–5.42)	2.62 (1.83–3.26)	0.685

Data are presented as mean ± standard deviation, median and interquartile range in round brackets and mean and range in square brackets, respectively.

**Fig 1:**
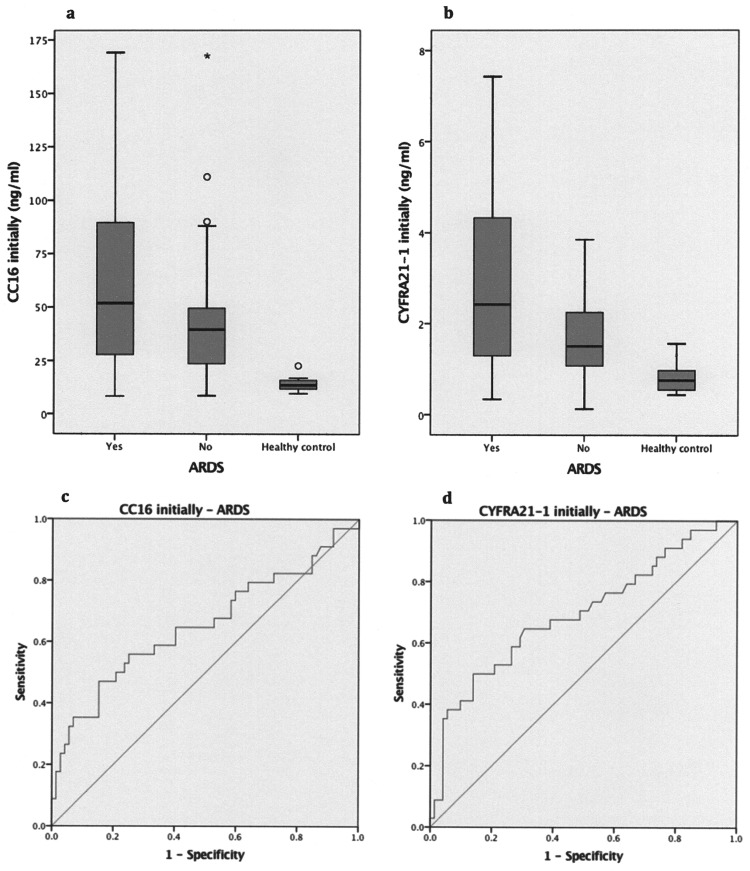
Biomarker levels and ARDS. Levels of **a.** CC16 and **b.** CYFRA21-1 assessed in polytraumatized patients with severe chest trauma immediately after admission and in healthy controls. **c.** ROC curve for initial CC16 level and ARDS. **d.** ROC curve for initial CYFRA21-1 level and ARDS.

Initial CC16 levels and CYFRA21-1 levels were higher in individuals with ARDS, as provided in Table 2 and graphically displayed in Fig 1a and 1b, whereas no significant differences remained on day 2 after the trauma.

Logistic regression analyses with each of the initial and day-2 level of sRAGE, CC16, SP-D, CYFRA21-1, and KL6/MUC1 as the single predictor variable and the occurrence of ARDS as the dependent binary variable respectively provided significant results solely for the initial level of CC16 (OR, 1.93; 95% CI, 1.08–3.47; p = 0.027) and the initial level of CYFRA21-1 (OR, 1.68; 95% CI, 1.09–2.60; p = 0.019). Single logistic regression analyses combining the biomarker levels assessed at the two time points as well as several multiple logistic regression analyses, including the combination of CC16 and CYFRA21-1, had less predictive power than the initial level of CC16 and CYFRA21-1 alone.

ROC statistics was performed for all five selected cytokines. The initial biomarker level and the day-2 biomarker level were used as the continuous variable and the occurrence of ARDS as the dichotomous variable. Table 3 shows that significant results were solely observed for the initial levels of CC16 and CYFRA 21–1 with CYFRA21-1 identified as best predictor according to the highest AUC.

**Table 3:** ROC statistics for biomarker levels and the occurrence of ARDS.

	At admission			Post-injury day-2		
	AUC	95% CI	p	AUC	95% CI	p
sRAGE	0.584	0.468–0.791	0.169	0.619	0.490–0.749	0.064
CC16	**0.628**	**0.501–0.754**	**0.038**	0.561	0.432–0.691	0.340
SP-D	0.500	0.380–0.620	0.997	0.568	0.441–0.696	0.288
CYFRA21-1	**0.674**	**0.556–0.792**	**0.005**	0.546	0.420–0.671	0.483
KL-6/MUC1	0.625	0.411–0.839	0.281	0.518	0.287–0.748	0.880

Bold characters denote significant results.

The relevant ROC curves are presented in Fig 1c and 1d, with a cut-off value of 47.94 ng/ml (sensitivity, 55.9%; specificity, 70.8%) for initial CC16 levels and a cut-off value of 1.85 ng/ml (sensitivity, 76.7%; specificity, 66.2%) for initial CYFRA21-1 levels.

### Pneumonia

**Fig 2:**
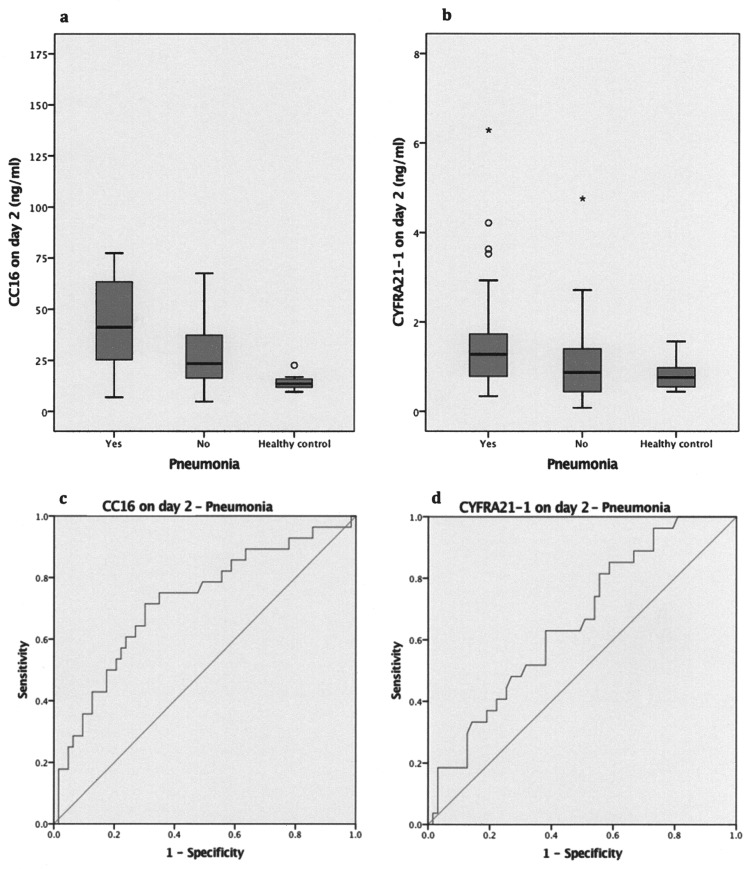
Biomarker levels and pneumonia. Levels of **a.** CC16 and **b.** CYFRA21-1 assessed in polytraumatized patients with severe chest trauma on day 2 after trauma and in healthy controls. **c.** ROC curve for day-2 CC16 level and pneumonia. **d.** ROC curve for day-2 CYFRA21-1 level and pneumonia.

Pneumonia as a complication of the trauma developed in 30 patients (29.7%). It was caused by several pathogens (S1 Table). First signs of pneumonia were clearly distinct on day 4 after admission. In all individuals with pneumonia and ARDS, pneumonia was diagnosed after the occurrence of ARDS. Patients with pneumonia presented a higher overall thoracic injury severity and they had to be ventilated longer than those patients without pneumonia, resulting in a longer ICU LOS (Table 2). Pre-hospital intubation and pre-hospital chest-tube insertion have been identified as significant risk factors for the occurrence of pneumonia. Notably, levels of CYFRA21-1 and CC16 were higher in patients with pneumonia at admission and on day 2 after trauma, as shown in Table 2. Boxplots referring to CC16 levels and CYFRA21-1 levels assessed on day 2 and healthy controls are presented in Fig 2a and 2b.

Logistic regression analyses were performed with each of the initial and day-2 level of sRAGE, CC16, SP-D, CYFRA21-1, and KL6/MUC1 as single predictor variable and the event “occurrence of pneumonia” as binary dependent variable, obtaining significant results for the initial CC16 level (OR, 2.21; 95% CI, 1.18–4.12; p = 0.013), the initial CYFRA21-1 level (OR, 1.83; 95% CI,1.15–2.89; p = 0.010), the day-2 CC16 level (OR, 2.68; 95% CI, 1.28–5.59; p = 0.009), and the day-2 CYFRA21-1 level (OR, 1.91; 95% CI, 1.08–3.36; p = 0.026). Single logistic regression analyses combining the biomarker levels assessed at the two time points as well as multiple logistic regression analyses, including the combination of CC16 and CYFRA21-1, had less predictive power than each of the initial and day-2 levels of CC16 and CYFRA21-1 alone.

AUCs were computed for all five selected cytokines and the occurrence of pneumonia. Table 4 reveals significant results for both the initial and follow-up levels of CC16 and CYFRA21-1. Follow-up CC16 was identified as the best predictor of pneumonia by means of the highest AUC.

**Table 4:** ROC statistics for biomarker levels and the occurrence of pneumonia.

	At admission			Post-injury day-2		
	AUC	95% CI	p	AUC	95% CI	p
sRAGE	0.464	0.344–0.583	0.571	0.578	0.452–0.705	0.236
CC16	**0.656**	**0.530–0.783**	**0.015**	**0.721**	**0.604–0.839**	**0.001**
SP-D	0.423	0.302–0.544	0.230	0.474	0.345–0.604	0.699
CYFRA21-1	**0.692**	**0.572–0.811**	**0.003**	**0.658**	**0.540–0.775**	**0.018**
KL-6/MUC1	0.493	0.286–0.700	0.958	0.554	0.276–0.831	0.678

Bold characters denote significant results.

The corresponding ROC curves referring to the day-2 levels of CC16 and CYFRA21-1 in polytraumatized patients suffering a severe chest trauma with and without pneumonia are presented in Fig 2c and 2d. Of CC16, 49.10 ng/ml (sensitivity, 58.6%; specificity, 75.7%) was determined as the cut-off value for its initial level and 30.51 ng/ml (sensitivity, 71.4%; specificity, 69.85) as the cut-off value for its day-2 level. The ROC curve based on the occurrence of pneumonia and the initial CYFRA21-1 levels provided a cut-off value of 2.49 (sensitivity, 55.2%; specificity, 81.4%), whereas a cut-off value of 1.26 (sensitivity, 51.9%; specificity, 68.3%) was revealed for the day-2 level.

Trauma victims, who developed both ARDS and pneumonia, presented median CC16 levels of 61.28 (25.85–115.13) ng/ml at admission and of 41.95 (21.29–65.94) ng/ml on day 2. Median CYFRA21-1 levels amounted to 3.55 (1.58–6.95) ng/ml on admission and to 1.11 (0.76–2.08) ng/ml on day 2. Moreover, CC16 levels were lower in survivors than in non-survivors [at admission, 40.35 (23.57–56.96) ng/ml versus 124 (78.96–154.78) ng/ml, p = 0.002; on day 2, 25.21 (17.42–42.13) ng/ml versus 457.58 (284.86–630.30) ng/ml; p<0.0001]. The same applies to CYFRA21-1 [at admission, 1.58 (1.12–2.49) ng/ml versus 16.76 (3.68–361.85) ng/ml, p = 0.001; on day 2, 0.91 (0.55–1.51) ng/ml versus 19.53 (6.29–32.76) ng/ml; p<0.0001]. Given the low number of deaths, logistic regression analyses and ROC curves would yield rather imprecise and possibly unreliable results and were thus not included in this paper.

## Discussion

Biomarkers capable of identifying trauma victims at risk for pulmonary complications would be of great help in clinical practice because their levels could be obtained early and objectively and are not subject to personal interpretation. Briefly summarized, our different statistical evaluations provided consistent results. CYFRA21-1 levels measured immediately after admission within one hour after the trauma, were identified as the most promising predictor of ARDS in polytraumatized patients with severe chest trauma, whereas CC16 levels assessed on day 2, (24 to 48 hours after trauma), have to be considered as most appropriate to predict pneumonia.

All five selected biomarkers are abundant in the lung, but they are not lung-specific. RAGE is expressed in almost all tissues of healthy adults [33]. CC16 is present in urogenital secretions [34]. SP-D is found in the lining epithelial cells in almost all exocrine ducts and the mucosa of the gastrointestinal and genitourinary tract [35]. CK19 is expressed in the lining of the gastroenteropancreatic and hepatobiliary tracts [36], and KL-6/MUC1 is found on epithelial cells that line the mucosal surfaces of the digestive system [37]. As presented in Table 1, levels of sRAGE, CC16, CYFRA21-1, and KL-6/MUC1 were two to three times higher in polytraumatized patients when assessed directly at admission within one hour after the trauma, compared to healthy controls, whereas SP-D levels decreased by one third. All of our polytraumatized patients suffered a severe chest trauma. Therefore, it can be safely assumed that tremendous mechanical forces had been applied to their thoraces and had been transmitted to all components of the thoracic cavity, resulting in intra-thoracic damage that corresponded to point of origin, direction and intensity of the external particular force. Moreover, most of our patients suffered serious injuries besides their severe chest trauma, as indicated by ISS values ranging from 17 to 59. The mechanical insult to the lung parenchyma and/or other body regions seems to cause an immediate release of sRAGE, CC16, CYFRA21-1, and KL-6/MUC1 into the circulation. Surprisingly, a reverse effect was observed for SP-D. Shortly after, follow-up processes start to decrease the level of each biomarker, providing significantly lower levels on day 2 compared to the relevant level at admission. With regard to ARDS and pneumonia, neither comparison of medians nor logistic regression analyses, or ROC statistics provided a statistically significant result for the initial and day-2 levels of sRAGE, SP-D and KL-6/MUC1, thus concordantly indicating that these three biomarkers were inappropriate to identify patients at high risk for pulmonary complications and therefore not included in the further discussion.

Unfortunately, the basic mechanism of leakage of CC16 and CYFRA21-1 into the circulation is hardly described in the available literature and totally lacking in the trauma setting. CC16 is a secreted product of the respiratory epithelium that is predominantly produced by the Clara cells of the distal respiratory and terminal bronchioles [38]. It diffuses passively across the alveolar-capillary barrier into the serum [39]. CK19, an acidic (type I) intermediate filament protein and therefore part of the cytoskeleton [40], is expressed in bronchial epithelial cells and in type I and type II alveolar epithelial cells [41]. Whereas its concentration is very low in healthy individuals [42], CK19 is over-expressed in many lung cancer tissue specimens [43]. During the transformation from normal to tumor tissue, CK19 is cleaved in neoplastically transformed epithelial cells because of increased protease activity of caspase 3 during apoptosis, and its soluble fragment CYFRA21-1 is released into the serum [43–45]. In patients with chronic airway inflammatory diseases, CYFRA21-1 is released in the BAL fluid by neutrophil elastase from bronchial epithelial cells and not from alveolar macrophages, neutrophils and fibroblasts [46]. Immunohistochemical analysis identified hyperplastic or injured type II alveolar epithelial cells as the main source of CYFRA21-1, detected in the BAL fluid of patients with ARDS [30].

With regard to ARDS, significant results were only found for the initial levels of CC16 and CYFRA21-1, with CYFRA21-1 providing the best result in any of the three statistical evaluations. CYFRA21-1 might be an early predictor of ARDS in polytraumatized patients with severe chest trauma. According to our findings CYFRA21-1 level exceeding the cut-off value of 1.85 would accurately identify 76.7% of polytraumatized patients developing ARDS, whereas 66.2% of the polytraumatized patients with an initial CYFRA21-1 level lower than 1.85 would actually not suffer post-traumatic ARDS. On day 2 CYFRA21-1 levels did not differ significantly between individuals with and without ARDS (Table 2). This finding can be explained by the fact that 75% of our patients developed ARDS by the second day. It is well known that ARDS is triggered by injury to the alveolar-capillary barrier. In severe chest trauma, the direct mechanical impact to the lung parenchyma and/or mediators of the early inflammatory immune response might be the cause for initial damage that leads to ARDS. Simultaneously, CYFRA21-1 might be separated from the cytoskeleton of epithelial cells as a result of cell injury and released into the serum in largest amounts due to increased permeability of the alveolar-capillary barrier. Our findings indicate that the leakage of CYFRA21-1 into the serum had remarkably decreased remarkably after the syndrome had emerged. An enhanced clearance by the kidneys within the first two days after the trauma may be one of the reasons for it.

With regard to pneumonia, comparison of medians, logistic regression analyses and ROC statistics provided significant results for the initial and day-2 values of CC16 and CYFRA21-1, identifying day-2 CC16 levels as the best predictor of pneumonia. This finding is consistent with the onset of pneumonia on day 4 or later. Our statistical analysis suggests CC16 levels, which are assessed on day 2 after trauma and exceed the cut-off value of 30.51, to accurately identify 71.4% of polytraumatized patients developing nosocomial pneumonia, whereas 69.85% of the individuals with a day-2 level of CC16 lower than 30.51 are expected not to suffer pneumonia. The primary task of CC16 is the defense of the respiratory tract against any pathogens that may invade the lung [47]. CC16 has anti-inflammatory and anti-oxidative properties [48, 49] because it inhibits phospholipase A2 activity [50] and chemotaxis of neutrophils and monocytes [51]. Furthermore, CC16 modulates lung inflammatory responses to infection, injury, and allergen challenge [52, 53] by downregulating pro-inflammatory cytokines including IFNγ, IL-1, IL-6 and TNFα [54]. TNFα is predominately expressed by monocytes and macrophages caused by a direct or indirect insult to the lung [55]. High levels of TNF-α have been detected in multiple injured patients in the early phase after trauma, ranging from four hours [56] to three days [57] post-injury. Mediated by TNFα host defense mechanisms get activated in response to an invading microbial pathogen [58] as is the case with nosocomial pneumonia even before the symptoms arise. Moreover, TNFα may modulate inflammatory responses in the airway by inducing CC16 expression [59]. To our opinion, the reported interaction between TNFα and CC16 might be the cause for significantly different levels of CC16 in polytraumatized patients developing and not developing pneumonia.

Unfortunately, the statistical significance of our results may not be robust enough to yield distinct information for the clinician. Until a clear conclusion can be drawn, more comprehensive trials have to be conducted, possibly focusing on injury patterns and comorbidities. CC16 and CYFRA21-1 levels assessed with the routine venous blood samples during primary survey and considered together with routine markers such as C-reactive protein levels and lactate levels might even increase their predictive power in clinical practice for identifying polytrauma victims with severe chest trauma at risk for ARDS and/or pneumonia.

## Conclusions

Our statistical analyses identified the initial level of CYFRA21-1 as the best predictor of ARDS, whereas the day-2 level of CC16 was considered most appropriate to predict pneumonia in polytraumatized patients with severe chest trauma. Basic research in order to evaluate the release mechanism of CYFRA21-1 and CC16 into the serum after severe chest trauma and the development of bedside tests would be helpful for optimal implementation of our findings in daily clinical routine. However, the actual benefit of CYFRA21-1 and CC16 cannot be known with certainty until studies are performed focusing on clinical decisions based on their serum levels.

## Supporting information

S1 Table Pathogens causing pneumonia. (DOCX)
