# Supplementary material for: Club cell protein 16 and cytokeratin fragment 21-1 as early predictors of pulmonary complications in polytraumatized patients with severe chest trauma
Source: PLoS One. 2017 Apr 5;12(4):e0175303. doi: 10.1371/journal.pone.0175303 (PMC5381917; doi:10.1371/journal.pone.0175303)
Supplement: S1 Table — (DOCX) [file pone.0175303.s001.docx]

| **Bacteria** | **Patients** |
| --- | --- |
| *Pseudomonas aeruginosa* | 7 (23.3%) |
| *Methicillin-sensitive Staphylococcus aureus* | 6 (20%) |
| *Enterococcus faecalis* | 5 (16.7%) |
| *Escherichia coli* | 3 (10%) |
| *Klebsiella pneumoniae* | 3 (10%) |
| *Methicillin-resistant Staphylococcus aureus* | 3 (10%) |
| *Enterobacter cloacae* | 1 (3.3%) |
| *Fungal pneumonia* | 1 (3.3%) |
| *Streptococcus pneumoniae* | 1 (3.3%) |

**S1 Table. Pathogens causing pneumonia**
